# Effectiveness of customized healing abutments in immediate implants: a randomized clinical trial

**DOI:** 10.1590/1807-3107bor-2025.vol39.084

**Published:** 2025-09-08

**Authors:** Mauro Dal Zot DUTRA, João Paulo DE CARLI, Felipe Gomes DALLEPIANE, Julia Cadorim FACENDA, Paulo Renato Pulga da SILVA, Yuri DAL BELLO, Letícia Copatti DOGENSKi, Marielle Bazzo DI DOMENICO, Pedro Henrique CORAZZA

**Affiliations:** (a) Universidade de Passo Fundo – UPF, School of Dentistry, Post-Graduation Program in Dentistry, Passo Fundo, RS, Brazil.; (b) Universdade Federal de Santa Catarina – UFSC, School of Dentistry, Post-Graduation Program in Dentistry, Florianópolis, SC, Brazil.

**Keywords:** Clinical study, Dental implants, Gingiva

## Abstract

This study evaluated the influence of a customized healing abutment (CHA) placed on immediate implants. It also assessed bone ridge volume, keratinized mucosal collar, and postoperative pain. Thirty-one patients needing tooth extraction and immediate implant were selected. Gingival papilla height, bone ridge volume, and keratinized mucosal collar thickness were measured using a millimeter periodontal probe and a dry-point caliper. A visual analog scale (VAS) was applied to determine postoperative pain. Study participants were randomly assigned to a control group (n = 15, with extraction, immediate implant placement, alveolar gap filling with a bovine bone graft, PTFE barrier, and suture) and an experimental group (n = 16, with extraction, immediate implant placement, gap filling with a bovine bone graft, and CHA). The healing abutments were prepared before surgery and customized during the procedure. The data were tabulated and statistically analyzed using the Mann-Whitney test (α = 0.05). The “changes in the papilla” outcome presented a statistical difference, and the control group showed higher values than those of the experimental group (p = 0.04). The remaining characteristics demonstrated no statistical differences between the groups (p > 0.05). CHA and the barriers exhibited similar behavior in maintaining bone ridge and keratinized mucosal thickness. CHA proved more effective than the conventional barrier in preserving the gingival papilla, with the distance measured three months post-surgery averaging 17% greater than the pre-surgical measurement.

## Introduction

In the presence of teeth, the periodontal ligament, periosteum, and bone tissue contribute to tissue vascularization. When tooth extraction or loss occurs, the periodontal ligament, as a source of blood supply, is lost, causing bone resorption in the early stages.^1^ The bone loses two-thirds of its volume in the first three months after extraction. Within two to three years, the healing and remodeling process eliminates 60% of the width and 40% of the height of the socket.^2^ Thus, implant placement immediately after tooth extraction is a common clinical practice. Immediate provisionalization would be the procedure of choice in these cases, recovering function and aesthetics and tamponading the surgical area.

Recent studies have highlighted that preserving the buccal bone wall is essential to prevent significant reductions in bone volume and ensure proper implant adaptation, improving long-term aesthetic outcomes.^3-5^ However, when this bone wall is compromised, bone regeneration becomes a significant challenge. The introduction of resorbable and non-resorbable barriers emerged as an alternative to protect the surgical site and minimize bone loss associated with tooth extractions. Among regenerative approaches, the use of non-resorbable polytetrafluoroethylene (PTFE) barriers has shown limited effectiveness. Although these barriers act as protective membranes, following the principle of osteopromotion,^6-8^ their combination with bone grafts is not always sufficient to maintain the necessary bone volume, with inconsistent results observed in terms of bone regeneration.^9^ These limitations underscore the need to develop more effective alternatives to optimize clinical outcomes, especially in cases of buccal bone resorption, ensuring both aesthetics and long-term implant stability.^10^

Although conventional healing caps vary in length, diameter, and shape, they primarily allow for soft tissue modeling to create a highly symmetrical architecture. Consequently, the use of provisional crowns is necessary to shape the peri-implant mucosa and progressively develop an optimal anatomical emergence profile before fabricating the definitive prosthetic crown. These steps are labor-intensive and may induce inflammation in the peri-implant tissues, potentially leading to tissue loss. Therefore, customized healing abutments are considered a superior alternative for guiding the appropriate architecture of the peri-implant mucosa compared to conventional healing caps.^11^

A customized healing abutment may represent an alternative to tamponade the surgical wound and maintain soft and hard tissue contours after immediate implant placement, positively influencing long-term implant health and simplifying treatment^9^ by avoiding a second surgical procedure. Additionally, this procedure might prepare the transmucosal area to receive the temporary or definitive crown during healing. Therefore, this study evaluated the influence of a customized healing abutment (CHA) placed on immediate implants in the posterior area over the gingival papilla. It also assessed bone ridge volume, keratinized mucosal collar, and postoperative pain. The tested hypothesis was that CHA would produce better outcomes than the PTFE barrier.

## Methods

### Study design and sample

This randomized clinical trial evaluated postoperative pain and tissue behavior in the posterior area of the maxilla and/or mandible in patients undergoing tooth extraction surgery and immediate osseointegrated implant placement. After this procedure, the alveolar gap was filled with a bovine bone graft (Bio-Oss Geistlich, Wolhusen, Switzerland), and the area was closed with a PTFE barrier (Lumina PTFE, Critéria^™^, São Carlos, Brazil) and suture (control group) or a customized CHA (experimental group) covering the surgical area and suture.

The study included 31 patients allocated to two groups: 15 in the control group and 16 in the experimental group), as described in a previous study.^10^ The required sample size, based on a power of 0.8 and an alpha level of 0.05, was five participants. After collecting data from five patients per group, the sample size was recalculated using the study’s own data, maintaining the same statistical power and alpha level, yielding a sample size of 14 participants. To account for potential losses, the sample size was set at 15 participants. The sample consisted of patients needing tooth extraction surgery and immediate osseointegrated implant placement treated at the dental clinics of the School of Dentistry of the University of Passo Fundo (DC/UPF), RS, Brazil, between 2021 and 2022. The study protocol was approved by the Research Ethics Committee of UPF (#48785421.2.0000.5342) and registered at the Brazilian Register of Clinical Trials platform (RBR-38c5f2h).

Patients who did not meet the preoperative or intraoperative criteria for immediate implant placement were excluded. as the criteria were absence of minimal bone availability to receive implants or surgical complications for tooth removal, requiring large buccal bone removal. Considering that insertion torque values in the range of 25–45 Ncm can prevent adverse micromovements (level between 50 μm and 100 μm) under loading, thus allowing for osseointegration.^12^ Implants with no primary stability and torque below 20Ncm were also excluded. Patients who did not follow the medication protocol recommended by the School of Dentistry and without gingival papillae were also removed from the study.

### Surgical procedure

The surgical procedures were performed by a team of residents in maxillofacial surgery and implant dentistry at DC/UPF under the guidance of the supervising professor. The primary researcher of the study (MDD) collected the data. Before the experimental procedures, MDD trained the professionals who performed the surgery. Interventions followed the standardized clinical approach adopted by DC/UPF.

Initial tests, a panoramic radiograph, and cone-beam computed tomography of the treatment area were performed. Preoperative tests included fasting blood glucose, complete blood count, coagulation profile, and vitamin D levels. Patients used the preoperative medication recommended by DC/UPF: amoxicillin 500 mg, four tablets taken one hour before surgery, or azithromycin 500 mg, one tablet taken one hour before surgery in cases of allergic patients. Intraoral antiseptic (0.12% chlorhexidine digluconate) was used for one minute before the procedure. Postoperative medication consisted of ibuprofen 600 mg taken every eight hours for three days and 0.12% chlorhexidine digluconate mouthwash for one minute, twice a day for 10 days. Immediate implant placement was performed after tooth extraction under local anesthesia (Alphacaine 100 - 2% lidocaine + 1:100,000 epinephrine, DFL, Rio de Janeiro, Brazil). The implants used were from Signo Vinces™ (Infra System, Campo Largo, PR, Brazil) and Neodent™ (CM System, Curitiba, PR, Brazil). The alveolar gap was filled with a bovine bone graft (Bio-Oss™, Geistlich, Woulhusen, Switzerland). Patients were then allocated to two groups, according to the method of socket tamponade.

### Groups

#### Control group – G1 (n = 15)

The cover screw provided by the manufacturer was installed over the implant. The alveolar gap was filled, and a barrier (Lumina PTFE, Critéria™, São Paulo, Brazil) was adapted and sutured. This barrier was removed after 21 days, following the manufacturer’s instructions.

#### Experimental group – G2 (n = 16)

The CHA was installed. This healing abutment consisted of a titanium UCLA (for temporary restorations) joined to a layer of photopolymerizable resin composite (Opallis™, FGM, Joinville, Brazil), forming a “plug” at the gingival level. The healing abutment was made according to the averages of mesiodistal (MD) and buccolingual/palatal (BL/BP) distances of molars and sterilized. The abutment was prepared during surgery according to each patient’s clinical situation, using rotary instruments. The healing abutments had three heights in the transmucosal area: 1.5, 2.5, and 3.5 mm.

## Randomization and blinding

A randomization list was generated using the random.org website (https://www.random.org/; control = 1 and experimental = 2). The operator was expected to have the PTFE barrier and the customizable healing abutment for each intervention, ensuring preparedness for the procedure in either group. The randomization list was transferred to individual, sealed, opaque (brown) envelopes that were passed on to the professor in charge of the clinic, each containing a letter indicating the assigned group. At the time of surgery and after implant placement, the patient drew one of the envelopes with the equivalent treatment. All clinical measurements were recorded by a calibrated examiner who was not involved in the surgical procedure. Patients were not informed about their treatments until the end of the study. The researcher responsible for data analysis was also blinded to group allocation.

## Outcomes

**Figure 1:**
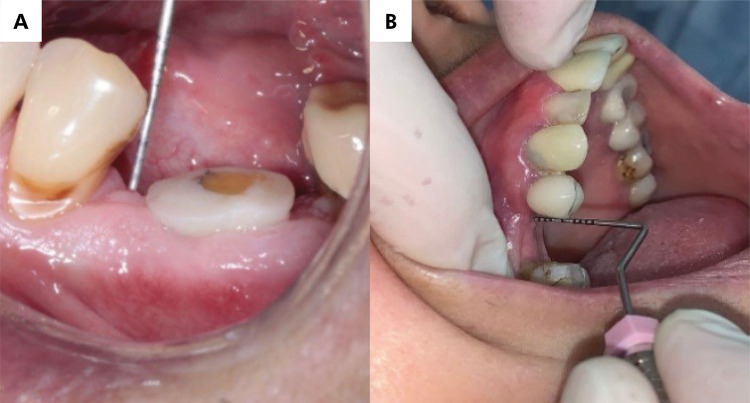
a) Measurement of the gingival papilla in the experimental group (from the mesial papilla to the marginal ridge of the neighboring tooth); b) Measurement of the mesial papilla position in the control group.

Clinical measurements for both the control and experimental groups were obtained before tooth extraction and after three months. The analysis used measurement differences before and after healing (Figures 1a and 1b). The statistical power (n = 15) was 0.48 for “changes in the gingival papilla,” representing the primary outcome of the study.

- Changes in the gingival papilla were evaluated by measuring the position of the mesial or distal gingival papilla up to an occlusal reference point (the marginal ridge of the neighboring tooth) using a millimeter periodontal probe.
- Bone ridge volume was assessed by measuring the buccopalatal/lingual width at the mucogingival junction with a thickness gauge (Figures 2a, 2b, and 2c). The statistical analysis was based on the difference between both measurements (day of surgery and second measurement after three months, on average).
  **Figure 2:**
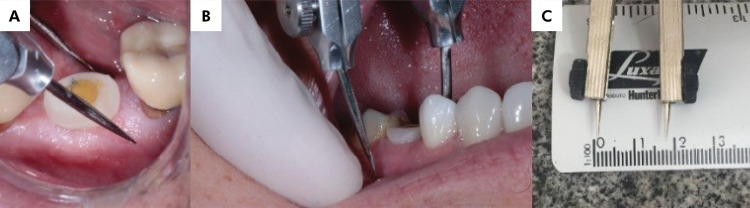
a and b) Measurement of bone ridge thickness with a dry-point caliper using the area of keratinized mucosa as reference; c)- Measurement recorded in millimeters.
- The height of the keratinized mucosal collar was measured from the mucogingival junction to the center of the tooth in question, using a millimeter probe. As in previous outcomes, the measurements were taken on the day of surgery and three months later, and the analysis was based on the differences between these two time points (Figures 3a and 3b).
  **Figure 3:**
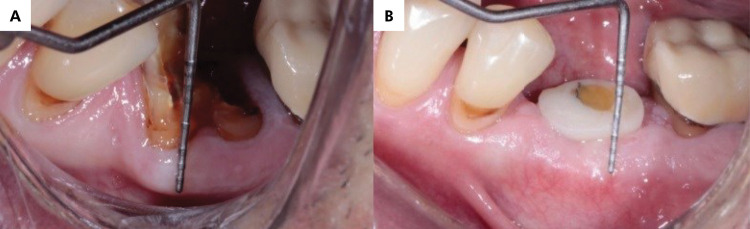
a) Measurement of the keratinized mucosa thickness before extraction using a millimeter probe; b) Measurement of the keratinized mucosal collar from the mucogingival junction to the crest of the alveolar ridge three months after implant placement.
- Postoperative pain was measured with a visual analog scale (VAS), determining pain intensity. The scale ranged from 0 to 10, where 0 indicated total absence of pain and 10 represented the maximum pain level bearable by patients. Four days after surgery, participants were asked to answer the following questions: Do you feel pain? How do you rate your pain?

## Statistical analysis

Considering the four outcomes, at least one study group did not follow a normal distribution (p < 0.05) according to the Anderson-Darling test (α = 0.05). Therefore, the Mann-Whitney test (α = 0.05) was used to compare the groups.

## Results

**Figure 4:**
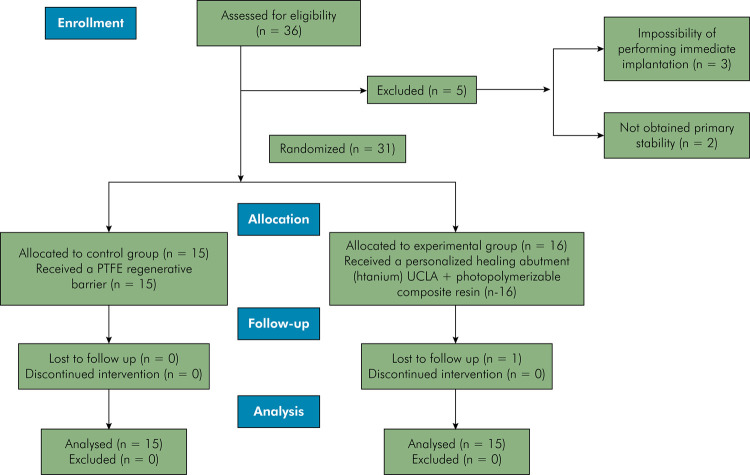
Consort flowchart of the study.

Out of the 36 eligible patients , five were excluded: three due to the impossibility of performing immediate implantation and two for not obtaining primary stability. Thirty-one patients were randomized: 16 into the experimental group and 15 into the control group. There was one loss to follow-up in the experimental group due to implant loss (implant success rate: 96.77%) (Figure 4).

Table presents the means, standard deviations, medians, Q1 and Q3 values, the comparison of gingival characteristics (including changes in the papilla and bone ridge volume), the thickness of the keratinized mucosal collar, and postoperative pain. Among all characteristics evaluated in the study, “changes in the papilla” was the only outcome to show statistical differences. The control group had higher difference values for the papilla than did the experimental group (p = 0.04). Other characteristics did not show statistical differences between the groups (p = 0.32 for alveolar ridge volume, p = 0.84 for keratinized mucosa, and p = 0.09 for pain).

**Table:** Number of participants, mean values before tooth extraction (mean before), mean values after three months (mean after), means of change (before - after)*, standard deviations (SD), minimum and maximum values, medians, Q1 and Q3 values, and the comparison of characteristics evaluated in the study.

Groups	n	mean _before_	mean _after_*	Mean ± SD*	Median	Q1	Q3	Comparison
Papilla								
Control	15	7.07	8.67	1.6 ± 1.35	2	1	3	p = 0.04
Experimental	15	7.37	8.14	0.77 ± 1.02	1	0	1	
Alveolar ridge volume								
Control	15	13.04	11.24	1.8 ± 1.42	2	0	3	p = 0.32
Experimental	15	12.6	11.38	1.22 ± 1.33	1	0	2	
Keratinized mucosa								
Control	15	3.9	2.64	1.26 ± 1.48	1	0	2	p = 0.84
Experimental	15	4.1	2.94	1.16 ± 0,91	1	0	2	
Pain								
Control	15	-	-	2.07 ± 2.17	2	1	2	
Experimental	15	-	-	2.5 ± 1.15	3	2	3	p = 0.09

*“Pain” was recorded solely on the fourth postoperative day.

## Discussion

A customized healing abutment for a given clinical situation may provide a correct gingival contour from the moment of implant placement surgery.^13^ This advantage alone provided sufficient rationale for conducting the present clinical trial. We opted for a clinical study design because of its close alignment with the routine practice of dental procedures. The hypothesis that CHA would allow for more favorable outcomes for the peri-implant gingival thickness of the keratinized mucosal collar and postoperative pain compared to the control group was partially accepted because the “changes in the papilla” outcome presented a positive statistical difference.

Finelle et al.^14^ conducted a study with 28 participants (14 received customized healing and 14 received xenogeneic collagen matrix) to evaluate the best method for tamponading the surgical wound after immediate implants. An optical scanner was used to collect the data and soft tissue anatomy images and to calculate volumetric changes. Digital models were exported from the scanner and superimposed at one, four, and 12 months of follow-up. Customized healing abutments generated a lower variation in the interdental papilla height than did xenogeneic barriers in the initial visual evaluation after the first month. Nonetheless, the final results of the study did not show statistical differences between CHA and the xenogeneic membrane groups because the groups did not show significant volumetric changes after one year.^14^

Finelle et al. stated that most peri-implant changes occur within the first month of evaluation. Canullo et al.^5^ support this statement in their study, which reported a more pronounced loss of bone width on the buccal side of the socket compared to the lingual and palatal sides after tooth extraction. Additionally, a loss of bone height was identified, although to a lesser extent than the horizontal contraction. The most significant changes occurred during the first month after extraction, after which bone remodeling was slower and stabilized by the fourth month. These findings highlight the rapid and inevitable bone remodeling in the first months post-extraction, emphasizing the importance of strategies to preserve bone volume and optimize outcomes in implant rehabilitations. The present study measured the outcomes only three months after implant placement, taking into account the early remodeling phase. Regarding the outcome of “changes in the gingival papilla,” most patients showed higher distance values after three months, as expected. The distance measured after three months was, on average, 17% higher than before surgery. As for the “bone ridge volume” outcome, an average decrease of 10.88% was observed. The reduction in the keratinized mucosal collar was 26%, regardless of the treatment. These findings are consistent with those of the referenced study.^14^

“Bone ridge thickness” and “keratinized mucosal collar” did not exhibit a significant difference between the control and experimental groups. Nevertheless, a case study conducted by Ruales-Carrera et al.^9^ emphasized the significance of CHA in helping to achieve a positive rehabilitation outcome. Also, Menchini-Fabris et al.^15^ reported that using CHA stabilizes the gingival architecture and bone volume in a fresh socket implant, while preserving the same emergence profile for restorative crowns, thereby eliminating the need for laboratory approximation of the emergence profile of the final restoration. Yet, the use of CHA contributes to optimal prosthetic-surgical planning and minimally invasive extraction by preserving supporting tissue integrity. This study aligns with our findings, indicating the advantages of using CHA.

A previous study,^16^ however, did not find benefits in CHA use. The authors assessed soft and hard tissue changes around immediate implants, comparing CHA with conventional industrial products in 28 immediately placed implants and the gap filled with substitute bone material. Group allocation was determined according to socket size: larger sockets using a customized healing abutment and smaller sockets using titanium. Radiographic and intraoral examinations were performed before and after surgery and at six months. Although customized abutments facilitate the closure of large alveoli, no statistical differences were observed between the groups. The study by Hu et al.^16^ has been criticized for evaluating sites of varying dimensions, potentially introducing a research bias.

de Carvalho Formiga et al. reported applying barriers to maintain the gingival architecture in post-extraction alveolar sites, with the advantage of minimizing bone loss in height and thickness.^17^ The findings of the present study concur with those of these authors. Conversely, bone ridge loss values in our study were higher (mean of 10.88% reduction) than those reported in their investigation, even when a 10.88% loss is deemed nonsignificant. Group comparisons demonstrate that CHA prevents a second-stage surgery for implant exposure providing more clinical time to make the final prosthesis and reducing tissue manipulation. An ideal transmucosal and prosthetic emergence profile development may also be based on the anatomy of the extracted tooth, facilitating molding procedures and promoting lower gingival pressure when installing the definitive crown.^18^ That was one of the best advantages of the present study because the transmucosal peri-implant area was almost ready for the definitive impression after three months.

The literature reinforces the importance of immediate implants in preserving gingival architecture and the stability of peri-implant soft tissues. de Siqueira et al. demonstrated that the immediate placement of implants combined with soft tissue grafting contributes to the maintenance of the gingival papilla and peri-implant esthetics, even after four years of follow-up.^19^ Furthermore, Maffei et al.^20^ compared different materials for alveolar sealing, such as free gingival grafts and collagen matrices, observing that both promote tissue preservation and a favorable environment for healing. These findings support the effectiveness of the customized anatomical healing abutment in the present study, which, by preventing a second surgical stage and facilitating impression procedures with less tissue manipulation, enhances the benefits of gingival papilla preservation and peri-implant esthetics observed in other approaches.

Postoperative pain did not generate significant differences between the control and experimental groups, using the VAS as a reference. The highest pain intensity reported by a patient in the control group was 8, and the lowest was 0; in the experimental group, the highest value was 4, and the lowest was 1. Most patients in both groups reported mild pain (1-3). Both groups followed the same pre- and postoperative protocols, including exams, pre- and postoperative medication, and postoperative care. The anesthetic and surgical techniques for extracting and placing the implants were also the same during surgery, with differences only in sealing the surgical site. Considering this characteristic, the hypothesis that postoperative pain is influenced by CHA was rejected.

Rizzatto et al. evaluated the postoperative pain of 108 patients treated at DC/UPF in 2018 and 2019. Postoperative pain was assessed using VAS scores , according to which most patients reporting mild pain (1–3). The most relevant factor for determining pain intensity was torque during implant placement. Patients with higher torques (50–80 N) reported higher postoperative pain intensity. Accordingly, the patient who reported severe pain (level 8 on the VAS) exhibited a primary stability value of 55 N.^21^ Therefore, the present study may relate pain to torque and not to the type of socket closure (membrane or customized healing device). It is also important to consider that primary stability with consequent new bone formation around the implant in immediate implants allows for more appropriate tissue healing,^22^ as observed in the present study.

Although it is recognized that implants have positive long-term effects,^23^ this clinical study posed some challenges. Part of the research was conducted during the COVID-19 pandemic, particularly in 2021, which hindered patient adherence and contributed to a relatively small sample size (n =31). Also, many cases had to be disregarded due to the exclusion criteria of “ absence of primary stability, with torque lower than 20 N.” This minimum torque for placing the healing abutment is sometimes hard to obtain in immediate implants. These were the limitations of our study. One dental implant in the experimental group was also lost one month after placement. Substantial biofilm and peri-implant mucosa with suppuration and bleeding were noted at implant removal, which might explain implant osseointegration failure.^24^

## Conclusion

The results showed that CHA and traditional barriers were similarly effective in maintaining the bone crest and the thickness of the keratinized mucosa, although CHA proved to be more effective than conventional barriers in preserving the gingival papilla. Additionally, CHA offers significant clinical advantages, particularly by simplifying treatment protocols and reducing the need for a second-stage surgery. These findings highlight the potential of CHA as a promising alternative to optimize aesthetic and functional outcomes in immediate implant treatments.

## Data Availability

The datasets generated during and/or analyzed during the current study are available from the corresponding author on reasonable request.
